# Structural brain network organization in children with prenatal alcohol exposure

**DOI:** 10.1016/j.nicl.2024.103690

**Published:** 2024-10-22

**Authors:** Xiaoyun Liang, Claire E. Kelly, Chun-Hung Yeh, Thijs Dhollander, Stephen Hearps, Peter J. Anderson, Deanne K. Thompson

**Affiliations:** a Murdoch Children’s Research Institute, Melbourne, Australia; b Florey Institute of Neuroscience and Mental Health, University of Melbourne, Melbourne, Australia; c Turner Institute for Brain and Mental Health, Monash University, Clayton, Victoria, Australia; d Department of Medical Imaging and Radiological Sciences, Chang Gung University, Taoyuan, Taiwan; e Department of Psychiatry, Chang Gung Memorial Hospital at Linkou, Taoyuan, Taiwan; f Department of Paediatrics, The University of Melbourne, Victoria, Australia

**Keywords:** Prenatal alcohol exposure, Diffusion MRI, Structural connectivity, Brain connectome

## Abstract

**Introduction:**

There is growing evidence suggesting that children with prenatal alcohol exposure (PAE) struggle with cognitively demanding tasks, such as learning, attention, and language. Complex structural network analyses can provide insight into the neurobiological underpinnings of these functions, as they may be sensitive for characterizing the effects of PAE on the brain. However, investigations on how PAE affects brain networks are limited. We aim to compare diffusion magnetic resonance imaging (MRI) tractography-based structural networks between children with low-to-moderate PAE in trimester 1 only (T1) or throughout all trimesters (T1-T3) with those without alcohol exposure prenatally.

**Methods:**

Our cohort included three groups of children aged 6 to 8 years: 1) no PAE (n = 24), 2) low-to-moderate PAE during T1 only (n = 30), 3) low-to-moderate PAE throughout T1-T3 (n = 36). Structural networks were constructed using the multi-shell multi-tissue constrained spherical deconvolution tractography technique. Quantitative group-wise analyses were conducted at three levels: (a) at the whole-brain network level, using both network-based statistical analyses and network centrality; and then using network centrality at (b) the modular level, and (c) per-region level, including the regions identified as brain hubs.

**Results:**

Compared with the no PAE group, widespread brain network alterations were observed in the PAE T1-T3 group using network-based statistics, but no alterations were observed for the PAE T1 group. Network alterations were also detected at the module level in the PAE T1-T3 compared with the no PAE group, with lower eigenvector centrality in the module that closely represented the right cortico-basal ganglia-thalamo-cortical network. No significant group differences were found in network centrality at the per-region level, including the hub regions.

**Conclusions:**

This study demonstrated that low-to-moderate PAE throughout pregnancy may alter brain structural connectivity, which may explain the neurodevelopmental deficits associated with PAE. It is possible that timing and duration of alcohol exposure are crucial, as PAE in T1 only did not appear to alter brain structural connectivity.

## Highlights

• Low-moderate alcohol exposure through gestation may alter brain networks at 6–8 years.
• Alcohol through gestation alters right cortico-basal ganglia-thalamo-cortical modules.
• Alcohol in trimester 1 only wasn’t associated with altered structural connectivity.

## 1 Introduction

The global prevalence rate of alcohol consumption during pregnancy is relatively high (∼10 %) (Popova et al., 2017), subjecting a large number of children to the possible adverse effects of prenatal alcohol exposure (PAE). Studies have shown that heavy drinking during pregnancy can result in fetal alcohol spectrum disorder (FASD) (Muggli et al., 2016). Children with FASD are commonly shown to have a broad range of neurodevelopment impairments including cognitive and learning deficits and behavioral and emotional challenges (Mattson et al., 2019). However, the effects of low-to-moderate PAE on neurodevelopment, especially the timing and duration of PAE (Guerri, 1998), remains unclear. Accumulating evidence has demonstrated that even low-to-moderate PAE may alter fetal development, leading to facial and neurological abnormalities and possibly poorer cognitive functioning (Lees et al., 2020, Long and Lebel, 2022, Muggli et al., 2017).

Aside from evidence of impaired neurodevelopmental outcomes in children with PAE, neuroimaging studies consistently show that these children exhibit a change in brain structure (Robertson et al., 2016, Zhou et al., 2018) or alteration of their brain networks (Fan et al., 2017). Such alteration of the brain network organization is thought to, at least in part, underlie cognitive dysfunctions (Menon, 2011, Sha et al., 2019). Magnetic resonance imaging (MRI) techniques are the most popular and powerful tool for mapping human brain networks, including functional connectivity (van den Heuvel and Hulshoff Pol, 2010) and structural connectivity based on diffusion-weighted imaging (DWI) (Yeh et al., 2021). The use of complex network analysis offers quantitative analyses of brain networks (Bullmore and Sporns, 2009), to investigate neurological disorders as well as the associations between them and behavioral outcomes (Fornito et al., 2015, Liang et al., 2021). Nevertheless, potential effects of PAE on brain network alterations have rarely been explored, and most studies focus on functional brain networks (Fan et al., 2017, Long and Lebel, 2022, Long et al., 2018). In a recent neonatal study, the effects of PAE were investigated on brain networks, both functionally and structurally (Roos et al., 2021). These studies have consistently demonstrated that PAE affects brain network organization, leading to brain function impairment (Fan et al., 2017, Long et al., 2018).

The aim of the present study was to compare brain structural connectivity of children with low-moderate PAE during trimester 1 only (PAE T1), children with low-moderate PAE throughout all trimesters (PAE T1-T3), and children with no PAE, at the levels of (a) the whole brain network using both network-based statistics (NBS) (Zalesky et al., 2010) and network centrality, (b) brain network modules, and (c) individual brain regions, including regions identified as hubs. We hypothesized that alterations of brain networks would be observed at all levels in both PAE T1 and PAE T1-T3 groups compared with the no PAE group. We also hypothesized that children exposed throughout gestation would have more altered structural brain networks compared with those exposed in trimester 1 only, based on known dose and timing dependent effects of PAE on brain development (Guerri, 1998).

## 2 Materials and methods

### 2.1 Participants

Asking Questions About Alcohol in Pregnancy (AQUA) is a longitudinal study of the neurodevelopmental outcomes in children with low to moderate PAE. The study originally recruited 1570 women and their offspring during early pregnancy from low-risk public maternity clinics in Melbourne between July 25, 2011 and July 30, 2012. Detailed information on the quantity and frequency of alcohol consumption was collected for T1-T3. Using previously described algorithms (Muggli et al., 2015, O'Leary et al., 2010), an equivalent absolute alcohol in grams (gAA) quantity was used to calculate a single-exposure measure for each stage of pregnancy by combining frequency, amount, and type of alcoholic drink. The PAE T1 group had 30 gAA per week on average, with 0.1 gAA per week on average post pregnancy recognition and included five with binge-level PAE prior to pregnancy recognition. The PAE T1-T3 group had 40 gAA per week on average prior to pregnancy recognition and <12 gAA per week throughout the remainder of pregnancy and included 11 with binge-level PAE prior to pregnancy recognition. A binge drinking episode was defined as ≥50 gAA per occasion. Those children whose mothers were abstinent from alcohol throughout pregnancy comprised the no PAE control group in the present study. We report on a cross-sectional brain imaging study as part of the larger AQUA project.

### 2.2 MRI data acquisition

In this study, 90 AQUA participants at ages between 6 and 8 years old were enrolled in the present study and underwent MRI, including 24 in the no PAE group, 30 in the PAE T1 group, and 36 in the PAE T1-T3 group. All mothers provided written consent prior to MRI scanning and protocols were approved by the Royal Children’s Hospital Human Research Ethics Committee. MRI data were collected on a Siemens 3T Prisma MR scanner (Erlangen, Germany). The following MRI data were collected: (1) DWIs were collected using a multi-shell acquisition scheme with 3 b-values (b = 750, 2000, and 2800 s/mm^2^) and associated volumes (25 (with 5 b = 0), 45 (with 5b = 0), and 60 (with 10b = 0), respectively), TR/TE = 3500/67 ms, flip angle = 90˚, matrix size = 124 × 124, 70 axial slices for whole brain coverage (2 mm isotropic), multiband acceleration factor = 2, parallel acceleration factor = 2; partial Fourier factor = 0.75. An additional set of b = 0 images with an opposing phase encoding direction (posterior-to-anterior and anterior-to-posterior) were collected for image distortion correction. (2) T1-weighted anatomical images (T1WIs) were acquired using the multi-echo magnetization-prepared rapid gradient echo (MP-RAGE) sequence (with echo-planar image-navigated prospective motion compensation) with the following parameters: TR = 2550 ms, TEs = 2.14/3.94/5.77/7.5 ms, flip angle = 8˚, matrix size = 288 × 288, 288 axial slices, voxel sizes: 0.9 × 0.9 × 0.9 mm^3^.

### 2.3 Data pre-processing

Image quality control was performed by visual inspection and those with severe movement artefact were excluded. Except where otherwise indicated, DWIs were processed using MRtrix3 version 3.0.2 (Tournier et al., 2019) with the following steps: (1) multi-shell DWI images were concatenated using “DWICAT” to account for intensity scaling variations across the separate scans; (2) data denoising using Marchenko-Pastur principal components analysis (Veraart et al., 2016); (3) Distortion and motion correction using Functional MRI of the Brain’s Software Library (FSL) TOPUP and EDDY tools with slice-to-volume correction (Andersson et al., 2003, Andersson et al., 2017), followed by visual inspection; (4) *B*_1_ bias field correction (Tustison et al., 2010); (5) Computing group-averaged response functions for brain white matter (WM), gray matter (GM), and cerebrospinal fluid (CSF) (Dhollander et al., 2019); (6) Computing fiber orientation distributions using multi-shell multi-tissue constrained spherical deconvolution (Jeurissen et al., 2014).

Anatomical T1WIs were processed as follows: (1) for each subject, the T1WI was registered to the corresponding DWIs by applying a rigid-body transformation using the flirt command of the FSL toolbox (https://fsl.fmrib.ox.ac.uk/fsl), with the processed mean b = 0 images as a reference; (2) tissue partial volume maps of GM, WM, and CSF were generated using the FSL toolbox (Patenaude et al., 2011, Smith, 2002, Zhang et al., 2001); (3) The default FreeSurfer version 7.1.1 reconstruction pipeline was applied to registered T1WIs using the Desikan-Killiany cortical atlas segmentation and excluding cerebellum, i.e. 82 regions were retained in the atlas. Then, the subcortical parts obtained with FreeSurfer parcellation were replaced by the subcortical GM partial volume maps generated in the step (2) (Smith et al., 2015a). Table 1 provides the list of final brain regions-of-interest or so-called network nodes considered in this study. The total intracranial volume (ICV) was estimated by using FreeSurfer (https://surfer.nmr.mgh.harvard.edu).

**Table 1:** Cortical and subcortical regions of interest.

Abbreviated brain region		Abbreviated brain region	
AC	accumbens	PaCG	paracentral gyrus
AM	amygdala	PHIG	parahippocampal gyrus
BSTS	banks of superior temporal sulcus	POP	pars opercularis
CACG	caudal anterior cingulate gyrus	POR	pars orbitalis
CMFG	caudal middle frontal gyrus	PTR	pars triangularis
CA	caudate	PCAL	pericalcarine cortex
CU	cuneus	PoCG	postcentral gyrus
EC	entorhinal cortex	PCG	posterior cingulate gyrus
FP	frontal pole	PrCG	precentral gyrus
FG	fusiform gyrus	PCU	precuneus
HI	hippocampus	PU	putamen
IPG	inferior parietal gyrus	RACG	rostral anterior cingulate gyrus
ITG	inferior temporal gyrus	RMFG	rostral middle frontal gyrus
IN	insula	SFG	superior frontal gyrus
ICG	isthmus cingulate gyrus	SPG	superior parietal gyrus
LOG	lateral occipital gyrus	STG	superior temporal gyrus
LOFG	lateral orbito frontal gyrus	SMG	supra marginal gyrus
LG	lingual gyrus	TP	temporal pole
MOFG	medial orbito frontal gyrus	TH	thalamus proper
MTG	middle temporal gyrus	TTG	transverse temporal gyrus
PA	pallidum		

A letter ‘L’ or ‘R’ is prefixed to the abbreviated region of interest to indicate left or right hemisphere in this article.

### 2.4 Construction of tractogram-based connectome

Whole-brain streamline tractograms were generated using the 2nd order integration over fiber orientation distributions (iFOD2) algorithm (Tournier et al., 2010) with the following parameters: step size = 1.25 mm, maximum curvature = 45° per step, length = 5–250 mm. Each tractogram of 10^7^ streamlines was obtained by seeding from GM-WM interface, tracking with the anatomically constrained tractography, and then post-processed using spherical-deconvolution informed filtering of tractograms (SIFT2) (Smith et al., 2015b) to correct for the connection density bias (Yeh et al., 2016). For each subject, structural connectomes were constructed by assigning the streamline weights obtained from SIFT2 to the closest node within a default radius (2 mm) of each streamline endpoint (Smith et al., 2015a). There was a total of 3,321 edges included in the connectivity matrix.

### 2.5 Brain network analysis

#### 2.5.1 Network modularity

**Fig. 1:**
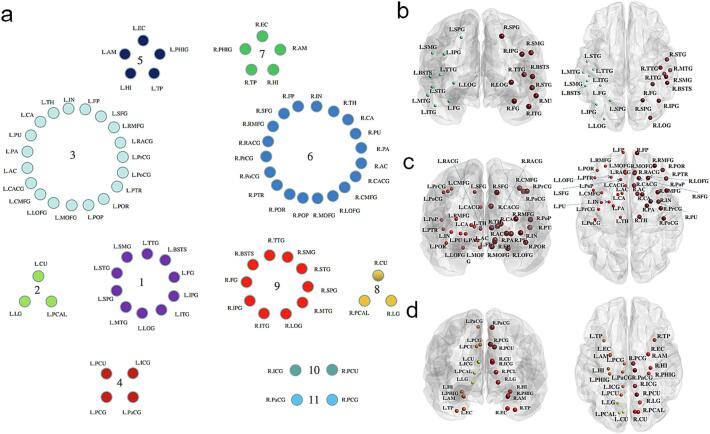
Network modules identified from 72 subjects from the 3 groups (i.e. 24 subjects per group) by using the consensus clustering approach. (a) Illustration of the 11 clusters identified. (b) The anatomical distribution of clusters 1 (small green nodes, left) and 9 (large dark red nodes, right). (c) Clusters 3 (small red nodes, left) and 6 (large dark red nodes, right). (d) Clusters 2 (small yellow nodes, left) and 8 (medium red nodes, right), 4 (small light orange nodes, left) and 10 & 11 (large dark red nodes, right), 5 (small dark orange nodes, left) and 7 (medial dark orange nodes, right). Note: Coronal view displayed on the left and axial view on the right. Brain region abbreviations are defined in Table 1. (For interpretation of the references to color in this figure legend, the reader is referred to the web version of this article.)

A common community structure was identified from whole brain data across all three groups using a consensus clustering approach, where edge weights were used to cluster brain regions into different communities with strong connections between nodes (Lancichinetti and Fortunato, 2012). For this purpose, 24 subjects were randomly chosen from the 3 PAE groups; this was intended to avoid potential bias due to an unbalanced sample size. Fig. 1 shows the common network community structure extracted across groups using this data-driven approach. A total of 11 modules were identified, including 5 clusters within the left hemisphere and 6 clusters within the right. Overall, these clusters were almost symmetrically distributed between left and right hemispheres, i.e. each cluster had a homotopic region on the contralateral side of it. However, a notable exception was cluster #4 within the left hemisphere, which corresponded to clusters #10 & #11 within the right hemisphere.

#### 2.5.2 Quantitative network analysis at different levels

Graph theoretical analysis was employed to quantitatively analyze brain networks by using the Brain Connectivity Toolbox (brain-connectivity-toolbox.net) at three levels: (i) At the whole-brain level: a global network measure was obtained by averaging all node-wise metrics; (ii) At the modular level: a modular measure was obtained by averaging node-wise metrics within a module; (iii) At the nodal level: a node-wise network metrics were computed for each subject.

In this study, we focused on network centrality, which is an indicator of the importance (i.e. how “central”) of a node within a network. Specifically, two centrality measures, i.e. the betweenness centrality (BC) and eigenvector centrality (EC), were used. Below are their definitions:

(i) BC is a measure of centrality based on the shortest paths in a graph. To identify the shortest path between a pair of nodes, the sum of the edge weights connecting the pair of nodes was minimized for the weighted network. BC for each node was calculated as the total number of the shortest paths that connect to the node.
(ii) EC considers that a node’s importance in a network is proportional to the sum of the importance of neighboring nodes in that network. Mathematically, the EC value for node *i* is defined as follows:
  $${\mathrm{EC}}_{i}=1/\lambda \sum_{j\in G}a_{i,j}{\mathrm{EC}}_{j}$$
  where *G* represents the other nodes in the network, $a_{i,j}$ denotes the edge weight in the adjacency matrix corresponding to nodes *i* and *j*, ${\mathrm{EC}}_{j}$ represents the EC of node *j*, and λ denotes the eigenvalue of the adjacency matrix. Therefore, a node is characterized by a high EC value if the node has direct connections to important nodes in the network.

However, the degree centrality was not considered due to its ambiguity in densely connected networks (Liang et al., 2019, Yeh et al., 2016).

#### 2.5.3 Summary measures of network metrics

Given that brain network properties depend on network density, it is common practice to apply a specific threshold to a target network. This results in either a sparse or dense network, though this process is somewhat arbitrary. To alleviate the issue of choosing arbitrary thresholds, a range of thresholds were employed to generate a series of networks covering network density from 5 % to 80 % with a step size of 5 %. The area under the curve (AUC) was then obtained by summing the values across the range of network densities, which is independent of any particular threshold.

#### 2.5.4 Hub brain regions extraction

(i) BC-based hub regions: A brain region with an AUC of BC (AUC-BC) greater than the mean AUC-BC plus one standard deviation was considered as a BC-based hub region.
(ii) EC-based hub regions: A brain region with an AUC of EC (AUC-EC) greater than the mean AUC-EC plus one standard deviation was designated as an EC-based hub region.

### 2.6 Statistical analyses

Between-group analyses were conducted to compare demographics and ICV between study groups. ANOVA models compared mean ages and ICV, and sex distributions were explored using chi-squared tests. All reported *p*-values are corrected for multiple comparisons, as detailed below.

#### 2.6.1 Network-based statistics

For edge-wise level statistical comparisons, NBS was used (Zalesky et al., 2010), which is a nonparametric approach that controls the family-wise error rate when involving multiple comparisons. Using NBS, two-sample one-tailed *t*-tests were conducted to detect the group differences in structural connectivity, controlling for age, sex, and ICV as confounding variables. Specifically, the NBS was conducted with a few different primary (*t*-statistic) thresholds between 2.1 and 3.0. PAE T1-T3 and PAE T1 were compared between each other, and both were tested against no PAE, the comparisons between any group pairs were made in either a positive or negative direction.

#### 2.6.2 Group-level statistical inferences of network metrics

For each network metric, either of whole-brain, modular, or nodal level, analysis of covariance (ANCOVA) was conducted by controlling for confounding variables, including age, sex, and ICV, with correction for multiple comparisons at each separate level using false discovery rate (FDR) at p < 0.05 level. Post-hoc tests comparing no PAE to PAE T1 and PAE-T1-T3 were performed with multiple comparison correction using Tukey’s honestly significant difference test.

#### 2.6.3 Statistical analysis of hub regions

For a node consistently identified as a hub across all three groups, statistical analyses were conducted to investigate whether there were significant differences in hub ranks, i.e. comparing hub structure, between PAE groups. Individual-level ranks for BC and EC were obtained by sorting the values, and the greatest value was considered having the highest rank. ANCOVA was conducted among groups to compare the hub ranks obtained either from the BC-based or EC-based approach, controlling for age, sex, and ICV. The multiple comparisons associated with each of the hub regions was corrected by controlling for FDR at p < 0.05 level.

## 3 Results

### 3.1 Participants

Participant characteristics are described in Table 2. The MRI data of 90 subjects were analyzed, including 24 no PAE, 30 PAE T1, and 36 PAE T1-T3. Sex differences were identified between the PAE T1 and no PAE groups (χ^2^(1, N = 54) = 5.7, p = 0.017), and between the PAE T1 and PAE T1-T3 groups (χ^2^(1, N = 66) = 5.3, p = 0.021). There were significant ICV differences among the PAE groups (p = 0.014), where PAE T1-T3 had significantly greater ICV than PAE T1 (p = 0.011), but there were no significant differences between the no PAE and either PAE T1 (p = 0.546) or PAE T1-T3 groups (p = 0.217). Age at assessment was similar between groups (p = 0.832).

**Table 2:** Participant characteristics.

		No PAE (*n* = 24)		PAE T1 (*n* = 30)		PAE T1-T3 (*n* = 36)	
		*n*	(%)	*n*	(%)	*n*	(%)
Child sex	*Female*	10	41.7	21	70.0	15	41.7
Child ethnicity	*White/Caucasian*	18	75.0	22	73.3	33	91.7
Child has any special health care needs	*CSHCN summary score^6^*	7	29.2	7	23.3	7	19.4
PAE binge episode	*Yes*	0	0	5	16.7	11	30.6
Maternal smoking during pregnancy	*Yes*	4	16.7	1	3.3	4	11.1
Maternal current smoking	*Yes*	1	4.2	0	0	2	5.6
Maternal education	*High school*	2	8.3	5	16.7	1	2.8
	*Trade/diploma*	6	25.0	6	20.0	6	16.7
	*Tertiary*	16	66.7	19	63.3	29	80.6
Family structure	*Nuclear, dual caregiver*	18	75.0	28	93.3	26	72.2
	*Separated, shared custody*	4	16.7	1	3.3	9	25.0
	*Sole parent*	2	8.3	1	3.3	1	2.8
Family financial situation	*Doing alright*	15	62.5	17	56.7	11	30.6
	*Living comfortably*	7	29.2	11	36.7	18	50.0
	*Finding it difficult*	2	8.3	2	6.7	7	19.4

		M	(SD)	M	(SD)	M	(SD)

Child age at MRI	*Years*	7.3	0.4	7.3	0.3	7.3	0.2
Child intracranial volume	*Cubic centimetres*	1556	139.4	1519	128.9	1613	121.5
Child IQ	*Composite score*	108.1	11.6	107.6	12.3	107.7	13.1
Maternal age at birth	*Years*	32.5	4.8	32.8	5.0	34.5	4.8
PAE T1, pre-pregnancy recognition	*absolute alcohol, grams/week*			29.6	89.8	40.2	49.4
PAE T1, post-pregnancy recognition	*absolute alcohol, grams/week*			0.1	0.3	7.3	21.7
PAE T2	*absolute alcohol, grams/week*					11.8	22.2
PAE T3	*absolute alcohol, grams/week*					8.0	12.5
Maternal current alcohol use	*AUDIT-C* s*ummary score*	2.4	2.0	2.7	1.7	3.5	1.9
General family functioning	*McMaster* s*ummary score*	1.5	0.4	1.5	0.4	1.7	0.5

PAE: prenatal alcohol exposure, T1: trimester one, T1-3: trimesters one to three, MRI: magnetic resonance imaging, CSHCN: Child Special Health Care Needs Screener (Bethell et al., 2002), M: Mean, SD: Standard deviation, IQ: intelligence quotient, measured using the Wechsler Intelligence Scale for Children (WISC-V Australian & New Zealand Standardised Edition) (Wechsler, 2014), AUDIT-C: Derived Alcohol Use Disorders Identification Test (Dawson et al., 2005), McMaster: McMaster Family Assessment Device; General family functioning sub-scale (Epstein et al., 1983).

### 3.2 Network-based statistical analyses

**Fig. 2:**
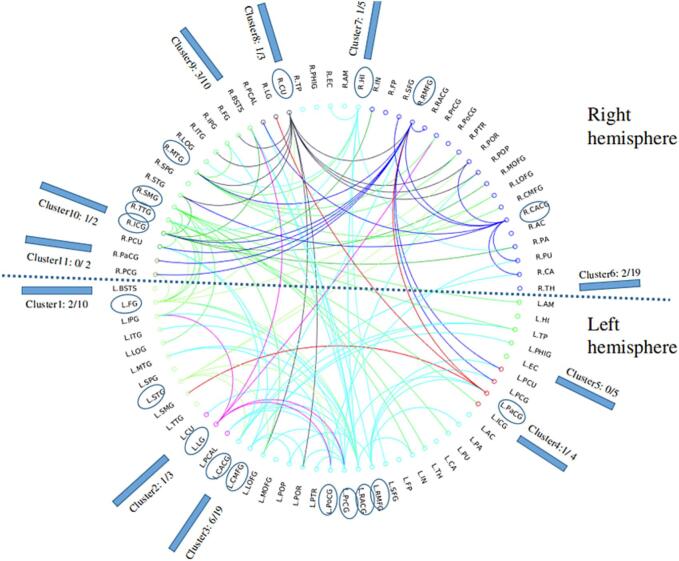
Identified altered network edges from the prenatal alcohol exposure throughout pregnancy group compared with controls using network-based statistics. A total of 126 decreased edges (66 nodes, Family Wise Error corrected p < 0.05) were identified from the prenatal alcohol exposure throughout pregnancy group when compared with the control group. For the purpose of visualizing brain regions with substantial alterations, regions with 5 or more altered edges are shown encircled with ellipses, and the number of these substantially altered regions within each cluster (module) is noted as a proportion of the total regions within that cluster. Note: the line colors are for visualization purposes, where the node belonging to the cluster with lower rank is considered as the starting node of one line; in this way, those lines that have the starting nodes within the same cluster will be assigned the same color. Brain region abbreviations are defined in Table 1.

As shown in Table 3, only *t* statistics between 2.1 and 2.5 were listed, as no significant differences were detected with any threshold above 2.5. The most significant difference (i.e., the lowest p-value of 0.038) between no PAE and PAE T1-T3 was identified at a threshold of *t* = 2.3. There were 126 significantly reduced network edge weights detected in 66 diffuse regions throughout the brain in the PAE T1-T3 group compared to no PAE (Fig. 2). Fig. 2 shows the 18 brain regions most heavily altered, each with five or more significantly reduced edge weights. These regions were distributed throughout the brain in frontal (bilateral rostral middle frontal gyri; left paracentral, precentral and caudal middle frontal gyri), temporal (right middle temporal and transverse temporal gyri; left superior temporal gyrus), limbic (bilateral caudal anterior cingulate gyri; right isthmus cingulate gyrus and hippocampus; left rostral anterior cingulate gyrus), occipital (right cuneus; left fusiform and lingual gyri) and parietal cortices (right supramarginal gyrus; left postcentral gyrus). No edges were significantly greater in PAE T1-T3 than in no PAE. No other group differences were observed.

**Table 3:** Network-based statistics results at different primary thresholds (no prenatal alcohol exposure vs. prenatal alcohol exposure throughout pregnancy).

Threshold	Nodes	Edges	*p*
2.1	72	183	0.042
2.2	69	150	0.042
2.3	66	126	0.038
2.4	63	92	0.05
2.5	−	−	0.0721

### 3.3 Network centrality analyses

#### 3.3.1 Whole brain level

There were no significant differences between groups in BC or EC.

#### 3.3.2 Module level

**Fig. 3:**
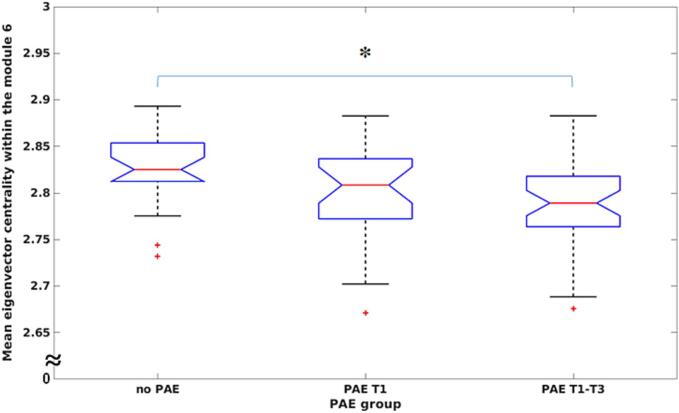
ANCOVA analysis of the mean eigenvector centrality by controlling for confounding factors age and sex for the three groups: prenatal alcohol exposure (PAE) in trimester 1 (T1) only; PAE throughout all trimesters (T1-T3); and no PAE.

Fig. 3 shows that a module (#6) in the PAE T1-T3 group had significantly lower EC (*p* < 0.027) than the no PAE group. Module #6 consisted of 19 regions in the right hemisphere; these included thalamus, insula, and basal ganglia (caudate, putamen, pallidum, and accumbens), the right frontal lobe (frontal pole, caudal middle frontal, lateral orbitofrontal, medial orbitofrontal, precentral, rostral middle frontal, and superior frontal gyri, pars opercularis, pars orbitalis, and pars triangularis), and also some regions of the limbic network (caudal anterior cingulate and rostral anterior cingulate gyri) and parietal lobe (postcentral gyrus) (see Fig. 1(d) and (e)). No significant PAE group differences were found in BC.

#### 3.3.3 Nodal level: Brain hub region analyses

**Fig. 4:**
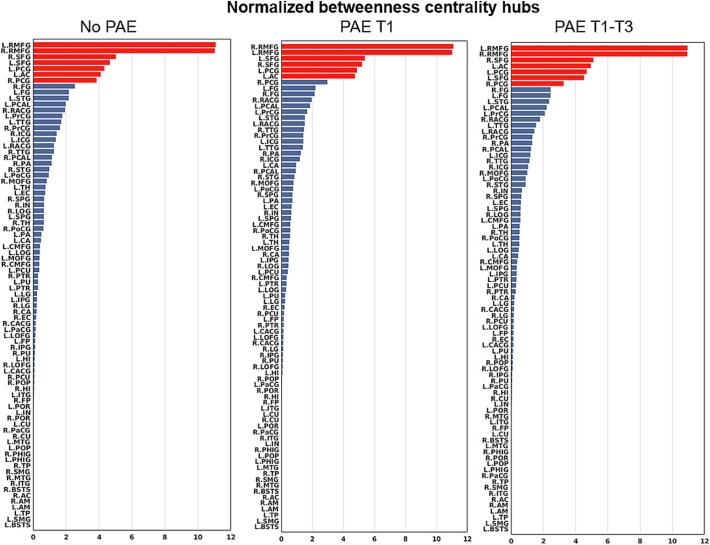
Bar graphs of normalized betweenness centrality (BC) of 82 brain regions in descending order calculated from the no prenatal alcohol exposure (PAE), the PAE in trimester 1 only (T1), and the PAE throughout all trimesters (T1-T3) groups. Normalized nodal BC of node i was calculated as BCi/mean(BC). Note: A brain region is identified as a hub region if its normalized betweenness is greater than the mean plus one standard deviation. Bars with red color represent those identified hub regions. Brain region abbreviations are defined in Table 1. (For interpretation of the references to color in this figure legend, the reader is referred to the web version of this article.)

**Fig. 5:**
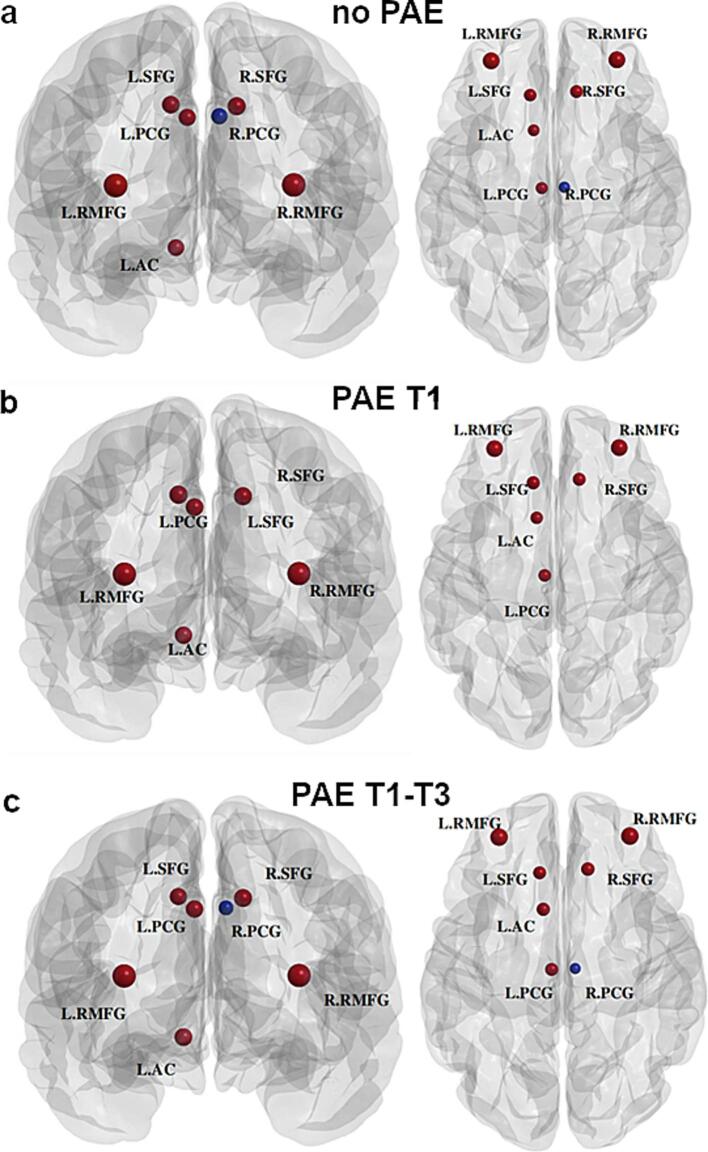
Visualization of hub regions identified based on betweenness centrality from structural brain networks for: (a) The no prenatal alcohol exposure (PAE); (b) The PAE in trimester 1 only (T1); and (c) The PAE throughout all trimesters (T1-T3) groups, displayed in coronal (left) and axial (right) views. Note: R.PCG was detected as a hub region for no PAE and PAE T1-T3 but not PAE T1, otherwise consistent hub regions were identified from each of the three groups. Brain region abbreviations are defined in Table 1.

Fig. 4 shows group-averaged node-wise BC values in a descending order, with hubs highlighted in red. The same regions were detected as hub regions (7 hubs) for the no PAE and PAE T1-T3 groups, including bilateral superior frontal, posterior cingulate, and rostral middle frontal gyri, as well as left accumbens. The PAE T1 group had the same hubs as the no PAE and PAE T1-T3 groups, except for the right posterior cingulate gyrus which was not identified as a hub region for PAE T1 (see Fig. 5). While the ranks of the hubs varied slightly, there were no statistically significant differences in hub structure (i.e. a set of hubs identified within the brain) among the three groups.

**Fig. 6:**
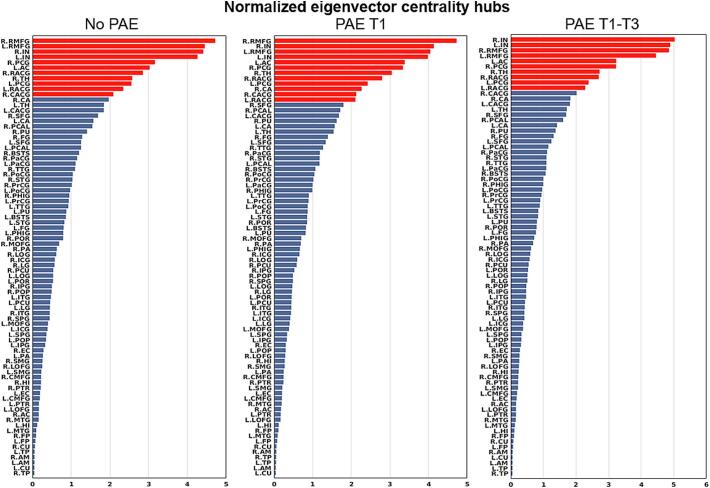
Bar graphs of normalized eigenvector centrality (EC) of 82 brain regions in descending order calculated from the no prenatal alcohol exposure (PAE) the PAE in trimester 1 only (T1), and the PAE throughout all trimesters (T1-T3) groups. Normalized nodal EC of node *i* was calculated as ECi/mean(EC). Note: A brain region is identified as a hub region if its normalized EC is greater than the mean plus one standard deviation. Bars with red color represent those identified hub regions. Brain region abbreviations are defined in Table 1. (For interpretation of the references to color in this figure legend, the reader is referred to the web version of this article.)

**Fig. 7:**
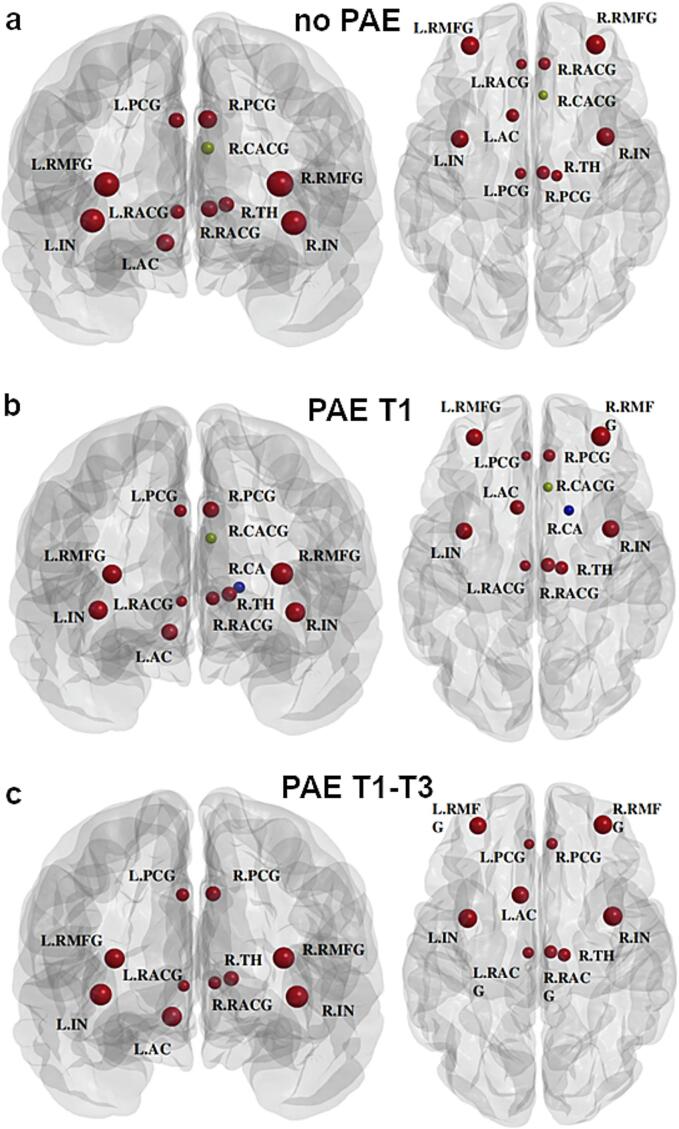
Visualization of hub regions identified based on eigenvector centrality from structural brain networks for: (a) The no prenatal alcohol exposure (PAE); (b) the PAE in trimester 1 only (T1); and (c) the PAE throughout all trimesters (T1-T3) groups, displayed in coronal (left) and axial (right) views. Note: R.CACG was detected as a hub region for both nPAE and PAE T1, whereas they were not identified as hub regions for the PAE T1-T3 group. Furthermore, R.CA was only detected as a hub region for the PAE T1 group. Brain region abbreviations are defined in Table 1.

Fig. 6 plots the group-averaged node-wise EC values in descending order, where 11, 12, or 10 hubs were extracted from the no PAE, PAE T1, and PAE T1-T3 groups, respectively. Fig. 7 shows the spatial distribution of these hubs, in which 10 regions were consistently identified as a hub in all the three groups, including bilateral posterior cingulate gyri, rostral middle frontal gyri, rostral anterior cingulate gyri, and insula, the right thalamus, and the left accumbens (Fig. 7). Additionally, the right caudal anterior cingulate gyrus was identified as a hub region for the no PAE and PAE T1 groups (Fig. 7(a, b)), and the right caudate was additionally detected as a hub region for the PAE T1 group only (Fig. 7(b). Despite some variations in EC values among those detected hub regions, no statistically significant differences in hub structure were observed between any of the groups.

#### 3.3.4 Nodal level: Individual brain regions

There were no significant differences of node-wise comparisons in BC or EC.

## 4 Discussion

### 4.1 Summary

Reduced structural connections between widespread brain regions were identified using NBS, and network alterations in EC were identified in a specific brain module resembling the right cortico-basal ganglia-thalamo-cortical network in children exposed to alcohol throughout pregnancy. However, there were no network- or module-level differences in those prenatally exposed to alcohol in T1 only compared with either controls or the PAE T1-T3 group. There were no PAE effects on connectivity centrality metrics noted at the brain regional level, including for brain hub regions.

### 4.2 PAE effects on network-based connectivity

The outcomes of NBS suggested decreased strengths of structural connections in children with low-to-moderate PAE throughout pregnancy, which can be interpreted as altered brain structural organization. This is consistent with a previous study which reported decreased structural WM connectivity in 5–18-year-old children with PAE at the whole-brain level (Long et al., 2020) which employed diffusion tensor imaging for constructing the structural connectomes. This reduced structural connectivity might underlie potential psychological, behavioral, and cognitive deficits in those with PAE (Lees et al., 2020). However, it should be noted that our evidence for network differences between PAE groups was weak, considering a lenient NBS threshold was used (*t* = 2.3; Table 3). Our results indicate the timing, duration, and overall dosage of PAE may be relevant, as there were no significant differences between the PAE T1 and no PAE groups.

### 4.3 PAE effects on network centrality: whole brain level

Network centrality comparisons at the whole-brain level did not reveal significant group differences, which is in contrast to a previous study showing reductions in related graph theory metrics, global efficiency, degree centrality, and participation coefficient, as well as increased shortest path length and betweenness centrality for 5–18 year olds with PAE (Long et al., 2020). The lack of significant differences for either BC or EC values between our PAE groups at whole-brain level likely suggests that the centrality metrics, measured as summary network metrics across the whole brain, may not be as sensitive as network edges in characterizing brain network alterations.

### 4.4 PAE effects on network centrality: module-level

A significantly decreased mean EC across all the brain regions within module #6 was identified for the PAE T1-T3 group, revealing that the brain regions within this cluster collectively have reduced connectivity with other important neighboring nodes in the network, compared with the no PAE group. No significant differences for BC were detected between PAE groups at the module level, likely implying that this measure of the total number of shortest path lengths passing through the node was not as sensitive as the EC measure, or the measure of shortest path length may not work well for structural connectomes (Civier et al., 2019). However, it should be noted that we cannot exclude the possibility that the significant difference of EC may be a false positive finding, which needs to be explored in future studies. Also relevant, is the possibility of false negatives, where there may have been reduced statistical power to detect significant group differences in smaller modules, as module #6 was one of the largest in size (19 nodes) of the 11 modules.

The EC-based module #6 involved the cortico-basal ganglia-thalamo-cortical network, including the corticostriatal and thalamocortical tracts that project to the frontal lobe. This may have implications for the cognitive and affective functions of those with low-to-moderate PAE in T1-T3, as well as somatosensory function given the involvement of the postcentral gyrus. The inclusion of the anterior cingulate gyrus in module #6 also insinuates alterations to widespread networks and brain functions, since it has connections with limbic, frontal, temporal, and parietal cortices, and is involved in many higher-level cognitive functions, impulse control, motivation, and emotion. Interestingly, significant findings occurred only in modules within the right hemisphere. There may be some lateralization of brain alterations associated with PAE, but this may also be explained by statistical power issues since NBS findings were not right-lateralized.

These EC-based findings are consistent with the results of NBS, in that most of the brain regions (15 out of 19) in module #6 were identified as being altered in the PAE T1-T3 compared with no PAE group. This highlights the distinction between connectivity analyses at different levels and using different techniques, and might imply that network centrality and NBS are complimentary. Indeed, the two techniques measure different aspects of connectivity: NBS is designed to identify significant edgewise differences between groups, while the module level network centrality analysis quantifies differences in summary network metrics. Furthermore, these results complement our recent findings that used cortical morphology and fixel-based diffusion imaging techniques to probe low-moderate PAE group differences in this cohort (Thompson et al., 2024). The study found that the right caudal anterior cingulate was smaller and had reduced surface area, and its white matter connection, the right cingulum bundle had reduced fiber cross-section in the PAE T1-T3 group compared with controls (Thompson et al., 2024). Given right caudal anterior cingulate connectivity was also altered in the current NBS analysis and was part of module #6 with altered network centrality in the TAE T1-T3 group, this highlights the teratogenic vulnerability of this important structure and its connections.

At the module-level, the presence of significant differences between PAE T1-T3 and no PAE groups and the absence of difference between the PAE-T1 and no PAE groups were consistent with the NBS findings. Collectively, these findings suggest that PAE beyond T1 may be required to alter the brain network connectivity. This contrasts with a recent study that revealed significant PAE effects on neonatal brain activity using electroencephalography, even when mothers quit drinking alcohol before Trimester 2 compared to a control group with no PAE (Shuffrey et al., 2020). Furthermore, significant PAE T1 effects have been demonstrated in an earlier study using the same cohort as the current study, in which associations between craniofacial shape and PAE were observed in the first trimester (Muggli et al., 2017). Thus, PAE only in T1 may be sufficient to cause adverse effects on offspring despite the findings of the current study, and we cannot conclude that PAE during T1 is ‘safe’. The lack of significant effects from the PAE T1 group in the current study could be multifactorial, and detecting any subtle alterations in PAE T1 may need a much larger sample size and other connectivity approaches, such as functional connectivity or cortical thickness-based structural connectivity.

### 4.5 PAE effects on network centrality: regional level

No significant differences in BC or EC between any groups were detected at the brain regional level. The inconsistent findings compared with significant differences detected in brain regions using the NBS technique and at the module-level suggest that network metric analysis at the regional level may not be sensitive enough to detect the subtle network alterations due to low-to-moderate PAE. Alternatively, our study could have lacked sufficient statistical power due to the small sample size relative to the number of brain regions investigated.

Although no statistical differences between PAE groups were found for brain hub structure, some insights could be gleaned from the hub analyses. In particular, most of the EC-based hub regions identified for the right hemisphere were also represented in module #6 (i.e. right rostral anterior cingulate and rostral middle frontal gyri, thalamus, and insula), which showed reduced average EC metrics in PAE T1-T3 compared with no PAE controls. The rostral anterior cingulate gyrus plays an important role in affect (Bush et al., 2000) and is an important component of the default mode network. The rostral middle frontal gyrus is involved in executive function, emotional regulation, and working memory, and has previously been identified as a connector hub (Gordon et al., 2018). The thalamus is an important relay station for information between subcortical areas and the cerebral cortex (Gazzaniga et al., 2002) and has been previously reported as a connector hub (Hwang et al., 2017). Being responsible for a wide range of functions from sensory and affective processing to high-level cognition, the insula is also recognized as an integrative hub region with a high level of connectivity within the brain (Gogolla, 2017). Additionally, we also identified the left rostral anterior cingulate and bilateral rostral middle frontal gyri hubs as heavily altered brain regions in the NBS analysis. Those with PAE have previously been shown to have significantly decreased brain volumes within the anterior cingulate (Aghamohammadi-Sereshki et al., 2022) and rostral middle frontal gyrus (Coles et al., 2011, Hendrickson et al., 2017). Thus, it could be that the connectivity of these hub regions is subtly or indirectly affected by low-to-moderate PAE, which might lead to functional deficits among this population. Further studies are required to confirm this finding.

Our converging findings suggest that low-to-moderate PAE may alter brain networks if exposure continues throughout pregnancy. While we may expect this to explain the poorer neurological functioning reported in those with PAE (Lees et al., 2020), not all studies have reported brain dysfunction, including our larger study for this cohort which reported no meaningful associations between low-to-moderate PAE and neurodevelopment at 6–8 years of age (Muggli et al., 2024). Likewise, another study showed no significant effects of low-to-moderate alcohol consumption on children’s behavior at 5 years old using the Strength and Difficulties Questionnaire (SDQ) (Skogerbo et al., 2013). Nevertheless, the authors inferred that the absence of statistically significant findings may be due to the difficulty in detecting subtle effects based solely on a single measure of neurodevelopment. In fact, the relatively low sensitivity of neurobehavioral measures has been recognized (Jacobson and Jacobson, 1994), and our findings indicate that structural connectivity analysis may be more sensitive for detecting subtle brain effects from low-to-moderate PAE. Indeed, it has been previously demonstrated that brain structural changes might be identified at lower levels of alcohol exposure than neurobehavioral deficits (Levin et al., 1992). Thus, it is possible that the altered networks we report are an indication of a biological effect of low-to-moderate PAE throughout pregnancy that is not strong enough to translate to poorer neurological functioning.

### 4.6 Strengths and limitations

Among structural connectivity studies focusing on PAE effects, most have employed the diffusion tensor imaging technique (Roos et al., 2021, Stephen et al., 2021, Taylor et al., 2015). However, diffusion tensor imaging cannot accommodate fiber crossing, bending, and twisting within voxels (Tuch, 2004). A strength of the present study is that we utilized multi-shell multi-tissue constrained spherical deconvolution tractography to address these issues (Tournier et al., 2019). Furthermore, the use of multi-shell DWI data (Jeurissen et al., 2014) and advanced quantitative tractogram processing techniques (Smith et al., 2012, Smith et al., 2015b) should lead to more reliable tractography results than conventional methods. Our study employed the current state-of-the-art pipeline to generate reliable structural brain networks, to ensure that our network analyses were conducted with the least bias possible (Yeh et al., 2021). The present study also has some limitations. Firstly, the smaller sample size precluded detailed PAE analysis, accounting for dose, pattern, and time of exposure (O'Leary et al., 2010). In the present study, only 3 groups were considered, which were classified only based on the time of exposure. Secondly, this study investigated PAE effects on brain network alterations based solely on structural connectivity. Multimodal MRI has already been shown to be more powerful in characterizing brain networks (Calamante et al., 2017, Calhoun and Sui, 2016), making it a more sensitive approach for detecting subtle PAE effects on brain network alterations in children subject to low-to-moderate PAE.

## 5 Conclusion

Our study provides new insights into the effect of low-to-moderate PAE during T1 only or throughout pregnancy on brain network alterations by using structural network analysis. While the effect of low-to-moderate PAE on brain development is thought to be subtle, this study confirms that low-to-moderate PAE throughout pregnancy could lead to alterations to major brain relay centers at both the network level and module-level. While the clinical implications of this study are yet to be determined, the findings support the avoidance of alcohol during pregnancy as the safest option.

## CRediT authorship contribution statement

**Xiaoyun Liang:** Writing – original draft, Visualization, Methodology, Formal analysis, Data curation. **Claire E. Kelly:** Writing – review & editing, Methodology, Data curation. **Chun-Hung Yeh:** Writing – review & editing, Visualization, Methodology. **Thijs Dhollander:** Writing – review & editing, Methodology, Data curation. **Stephen Hearps:** Writing – review & editing, Funding acquisition, Conceptualization. **Peter J. Anderson:** Writing – review & editing, Funding acquisition, Conceptualization. **Deanne K. Thompson:** Writing – review & editing, Funding acquisition, Conceptualization.

## Funding

This research was supported by the National Health and Medical Research Council of Australia (NHMRC) [Project Grant 1446635; Centre of Research Excellence in Newborn Medicine 1153176; Career Development Fellowship APP1160003 to DT; Investigator Grant APP1176077 to PA]; an Australian Government Research Training Program (RTP) scholarship and Monash University Graduate Excellence Scholarship to CK; the Murdoch Children’s Research Institute; the Royal Children’s Hospital Foundation; the Department of Paediatrics at The University of Melbourne; and the Victorian Government's Operational Infrastructure Support Program.

## Declaration of competing interest

The authors declare that they have no known competing financial interests or personal relationships that could have appeared to influence the work reported in this paper.

## Data Availability

Data will be made available on request.
